# Kainic acid Induces production and aggregation of amyloid β-protein and memory deficits by activating inflammasomes in NLRP3- and NF-κB-stimulated pathways

**DOI:** 10.18632/aging.102017

**Published:** 2019-06-10

**Authors:** Yang Ruan, Xiang Qiu, Yu-Dan Lv, Dong Dong, Xiu-Juan Wu, Jie Zhu, Xiang-Yu Zheng

**Affiliations:** 1 Department of Neurology and Neuroscience Center, The First Hospital of Jilin University, Changchun 130021, China; 2 Key Laboratory of Pathobiology, Ministry of Education, College of Basic Medical Sciences, Jilin University, Changchun 130021, China; 3 Department of Radiology, The First Hospital of Jilin University, Changchun 130021, China; 4 Department of Neurobiology, Care Sciences and Society, Karolinska Institute, Stockholm 141 86, Sweden

**Keywords:** kainic acid, NLRP3, NF-κB, interleukin-1β, brain-derived neurotrophic factor

## Abstract

Kainic acid (KA) treatment causes neuronal degeneration, which is a feature of Alzheimer’s disease (AD) symptoms such as amyloid β-protein production and memory deficits. Inflammasomes are known to be critical for the progression of AD. However, the underlying mechanism by which inflammasomes influence AD progression remains unknown. The present study investigated the damaging effect of KA on neurons by focusing on the inflammasome-mediated signaling pathways. Assessments using cultured microglia and mouse brains demonstrated that KA treatment specifically induced inflammasome activation. Mechanistic evaluations showed that KA activated two major components of inflammasomes, nucleotide binding oligomerization domain (NOD)-like receptor (NLR) protein 3 (NLRP3) and nuclear factor (NF)-κB, which resulted in the production of interleukin-1β (IL-1β) and brain-derived neurotrophic factor (BDNF). Inhibition of NLRP3 or NF-κB by Bay11-7082 caused a reduction in the KA-induced expression of interleukin (IL)-1β and BDNF. Moreover, knockdown of the expression of KA receptors (KARs) such as Grik1 and Grik3 induced suppression of NLRP3 and NF-κB, suggesting that KARs function upstream of NLRP3 and NF-κB to mediate the effects of KA on regulation of IL-1β and BDNF expression. Notably, IL-1β was shown to exert positive effects on the expression of BACE1, which is blocked by Bay11-7082. Overall, our results revealed that Bay11-7082 acts against KA-induced neuronal degeneration, amyloid β-protein (Aβ) deposition, and memory defects via inflammasomes and further highlighted the protective role of Bay11-7082 in KA-induced neuronal defects.

## INTRODUCTION

Kainic acid (KA), an analogue of the excitotoxin glutamate, can cause seizures in rodents and subsequent degeneration of selective populations of neurons in the hippocampus via subcutaneous or intraperitoneal administration, which is a model for studying neurodegeneration in humans [1, 2]. KA binds to specific excitatory amino acid receptors in the central nervous system and mediates certain nervous system diseases [3]. One example involves the excitotoxicity mediated by glutamate receptors, which underlies the pathology of a number of neuropathological conditions, including Alzheimer’s disease (AD), Huntington’s disease (HD), and Parkinson’s disease (PD). At the cellular level, experimental administration of KA was reported to typically induce excitotoxicity-related cell death, in which KA functions as a potent agonist affecting the α-amino-3-hydroxy-5-methyl-4-isoxazolepropionic acid (AMPA)/kainate class of glutamate receptors [4–6]. At the rodent level, injections of KA were reported to cause recurrent seizures, behavioral changes, and subsequent selective degeneration of neuronal populations in the brain [7, 8].

Microglial activation and astrocytic proliferation are the other characteristics of KA-induced neurodegeneration. The underlying mechanism mainly involves the critical regulatory function of KA in inducing the secretion of several cytokines in the respective diseases and modifying certain disease outcomes [9]. Specifically, several cytokines and inflammatory mediators secreted by activated glial cells, such as interleukin (IL)-1β, tumor necrosis factor (TNF)-α, and IL-6, are upregulated in the AD brain. Moreover, long-term exposure to these inflammatory cytokines leads to cognitive impairment during the course of AD development [10]. One example is the inflammatory cytokine IL-1β, the expression of which was known to be enhanced in KA-stimulated microglial cells, indicating the potential contribution of this cytokine in mediating the role of KA in the pathogenesis of AD [9]. Consistent with this speculation, KA was recently identified to be responsible for inducing learning and memory deficits in various neurodegenerative diseases [11]. After KA administration, rodents exhibited behavioral changes in their performance in a water maze, object exploration tasks, and the open-field test along with selective damage to the hippocampus. Deficiencies in short-term and long-term spatial learning were both observed [12].

Given the aforementioned potential contribution of KA to inflammation, it is easy to speculate if KA participates in activating inflammasomes and subsequently modulates the progression of AD. Although there is still no direct evidence to support such a hypothesis, the role of inflammasomes in AD pathology has been recently identified [13]. For instance, Aβ can activate the NLRP3 inflammasome in microglia [14], which is fundamental for the secretion of pro-inflammatory cytokines and subsequent inflammatory events. More importantly, activation of the NLRP3 inflammasome has demonstrated a critical role in AD pathogenesis by mediating harmful chronic inflammatory responses whereas inhibition of NLRP3 largely protected the loss of spatial memory and decreased Aβ deposition in an AD mouse model [13]. In addition, canonical activation of the NLRP3 inflammasome results in the assembly of different scaffold components: the cytoplasmic receptor NLRP3, the adaptor protein ASC, and the effector protein caspase-1. This association leads to the activation of caspase-1, inducing the maturation and secretion of IL-1β and IL-18, pyroptotic cell death, and unconventional secretion of cytokines and growth factors [15]. As a wide array of molecules induce the activation of NLRP3 inflammasome, its activation is tightly regulated at multiple levels, such as NF-κB [16]. Thus, it would be interesting to elucidate the roles of KA in regulating AD via inflammasome activation.

## RESULTS

### Kainic acid treatment activates the inflammasome in the brains of APP23 mice

**Figure 1:**
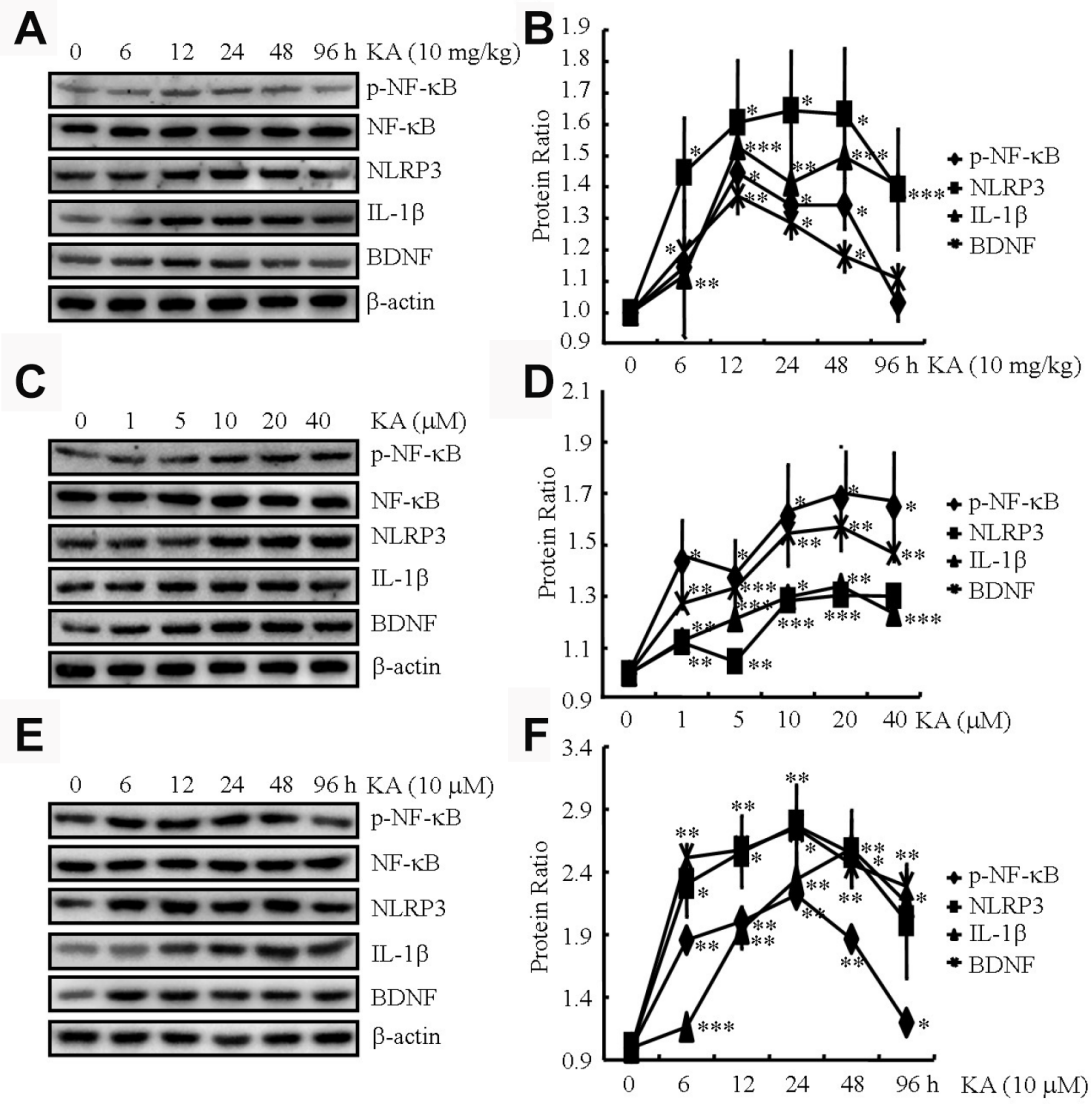
**KA augments the activity of inflammasome in vivo and in vitro.** (**A** and **B**) Phosphorylation levels of NF-κB and expression levels of NLRP3, IL-1β, and BDNF in KA-treated mice brains at different time points (N=6). (**C** and **D**) Phosphorylation levels of NF-κB and expression levels of NLRP3, IL-1β, and BDNF in KA-treated BV2 cells at different concentrations (N=3). (**E** and **F**) Phosphorylation levels of NF-κB and expression levels of NLRP3, IL-1β, and BDNF in KA-treated BV2 cells at different time points (N=3). The optical density of bands in Western blotting was analyzed by ImageJ software ^*^P<0.05, ^**^P<0.01, and ^***^P<0.001 vs the controls; the significant difference from the respective values were determined by the one-way analysis of variance test.

KA has been known to cause status epileptics, neurodegeneration, and memory loss [17]. To validate the neuronal toxicity of KA, we injected (i.p.) the APP23 mice with KA (10 mg/kg) for 6, 12, 24, 48, and 96 h and measured the phosphorylation of NF-κB and expression of NLRP3, IL-1β, and BDNF in the brains of the mice. At 12, 24, and 48 h after KA treatment, we observed a significant increase in the phosphorylation of NF-κB and the expression of NLRP3, IL-1β, and BDNF in the brains of the respective APP23 mice (Figure 1A and 1B). At 96 h after KA administration, the changes began to return to the control levels (Figure 1A and 1B). Thus, we harvested the brain tissue at 48 h after KA treatment for the experiments.

We further assayed the phosphorylation of NF-κB and the expression of NLRP3, IL-1β, and BDNF in microglial BV2 cells. At 48 h after KA treatment, NF-κB phosphorylation significantly increased in the BV2 cells, reaching the highest point at 10 μM (Figure 1C and 1D). In addition, the expression of NLRP3, IL-1β, and BDNF was elevated in the KA-treated BV2 cells **(**10 μM, Figure 1C and 1D**)**. Moreover, we treated the BV2 cells with 10 μM KA for the indicated time intervals. The results showed that KA started to induce the phosphorylation of NF-κB and the expression of NLRP3, IL-1β, and BDNF at 6 h, which was sustained to 48 h (Figure 1E, 1F). To maintain consistency between the in vitro and in vivo samples, we used 10 μM KA for 48 h in our subsequent assays. Taken together, our observations indicated that KA treatment could efficiently enhance the phosphorylation of NF-κB and the expression of NLRP3, IL-1β, and BDNF both in vivo and in vitro.

### NF-κB and NLRP3 colocalized with amyloid plaques

**Figure 2:**
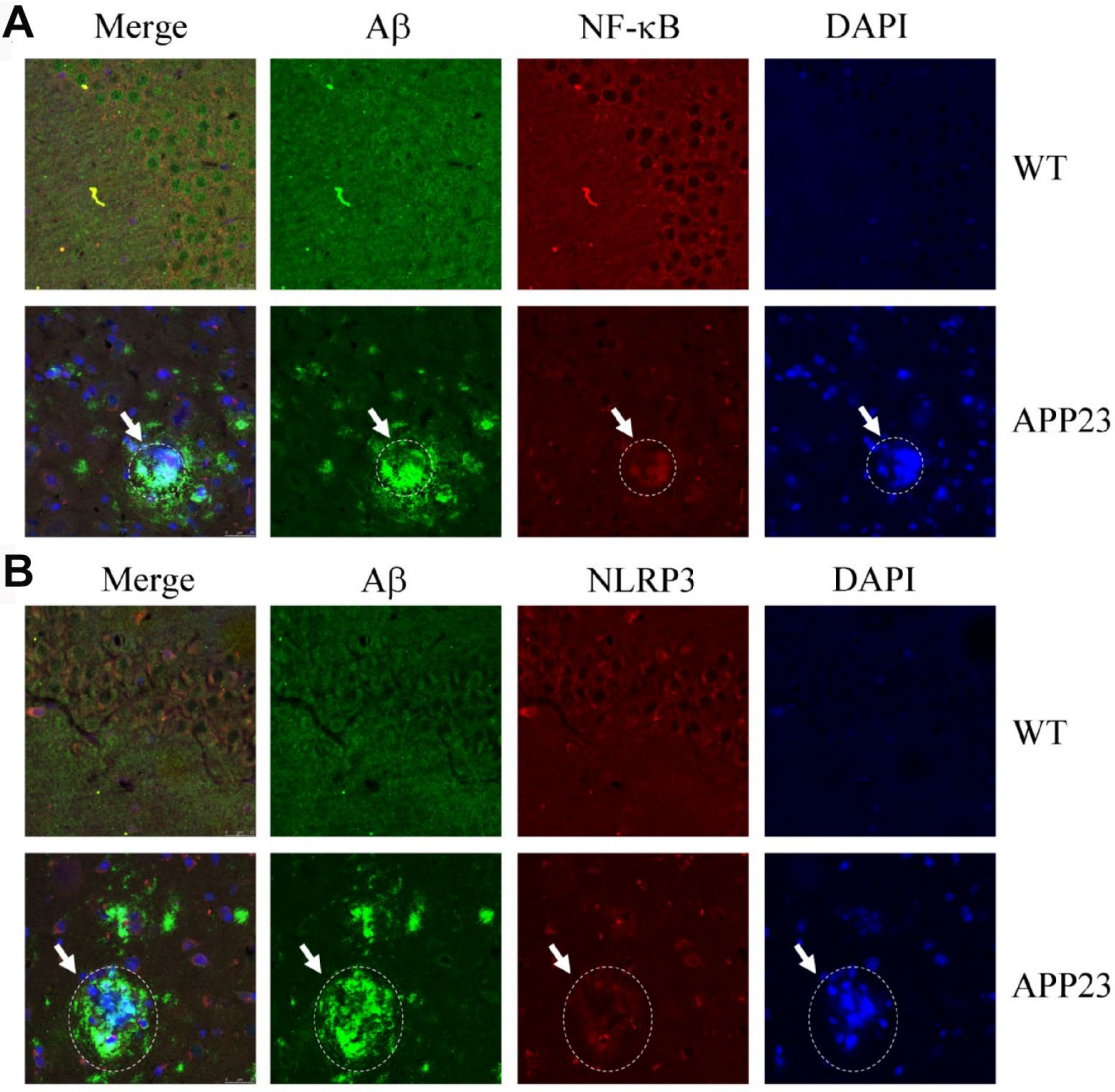
**Confocal fluorescence microscopy shows colocalization of NF-κB, NLRP3, and Aβ42 in the brains of APP23 mice.** Images from a single z-plane of the brains of APP23 mice at 6 months of age using monoclonal antibodies against Aβ (green), NF-κB/NLRP3 (red), and nucleus (blue DAPI stain). (**A**) Colocalization of NF-κB and Aβ42 is observed as indicated in the circle. (**B**) Colocalization of NLRP3 and Aβ42 was observed as indicated in the circle. Scale bar=25 μm.

Given the potential contribution of inflammasomes to AD [13], we characterized the relationship between NF-κB/NLRP3 and amyloid plaques (APs). Accordingly, confocal microscopic analyses were performed to study the allocation of NF-κB/NLRP3 and Aβ in 6-month-old APP23 mice (Figure 2). NF-κB was deposited with APs (Figure 2A). Similarly, NLRP3 was also aggregated with the APs (Figure 2B). These results clearly indicate that NF-κB and NLRP3 colocalized with APs.

### Grik1 and Grik3 mediate KA-induced phosphorylation of NF-κB and the expression of NLRP3, IL-1β, and BDNF

**Figure 3:**
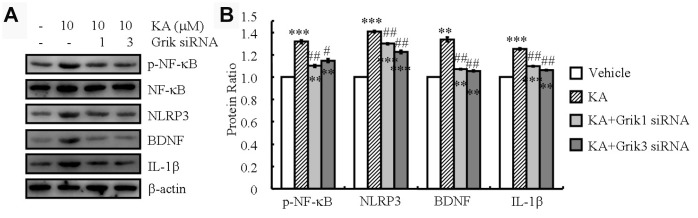
**Grik1 and Grik3 mediate the KA-induced phosphorylation of NF-κB and the expression of NLRP3, IL-1β, and BDNF.** Grik1 and Grik3 siRNAs (200 ng) were transfected to BV2 cells before treatment with KA (10 μM) (N=3). (**A**) Phosphorylation levels of NF-κB and expression levels of NLRP3, IL-1β, and BDNF were determined by Western blotting in KA and/or Grik1/3 transfected BV2 cells. (**B**) The optical density of bands in Western blotting was analyzed by ImageJ software ^**^P<0.01; ^***^P<0.001 vs the control group; ^#^*P*<0.05; ^##^*P*<0.01 vs the KA-only treatment.

KA receptors (KARs) are encoded by a set of separate genes (Grik1-3, KA-1, and KA-2) that are widely distributed throughout the brain [18]. They have been implicated to participate in epileptogenesis and cell death [18]. To assess whether KARs could exacerbate the KA-induced inflammasome, we performed siRNA interference experiments to assay the roles of KARs in the phosphorylation of NF-κB in BV2 cells. We observed that the mean level of NF-κB phosphorylation in the KA-treated cells (10 μM) increased compared to that in the control group. Moreover, phosphorylation of NF-κB was significantly attenuated in the KA-treated Grik1 and Grik3 knocked-down cells than in the KA-treated cells (Table 1). As shown via Western blotting, knockdown of Grik1 and Grik3 also decreased the stimulating effects of KA on the expression of NLRP3, IL-1β, and BDNF in the BV2 cells (Figure 3A and 3B). Taken together, these results demonstrated that Grik1 and Grik3 mediated the effects of KA by stimulating the phosphorylation of NF-κB and the expression of NLRP3, IL-1β, and BDNF in the BV2 cells.

**Table 1:** The effects of KA receptors on the phosphorylation of NF-κB in BV-2 cells.

	**KA**	**KA/Grik1**	**KA/Grik2**	**KA/Grik3**	**KA/KA1**	**KA/KA2**
p-NF-κB	1.32±0.16	1.10±0.01	1.22±0.10	1.15±0.02	1.13±0.10	1.20±0.09
Statistics	***	##	NS	#	NS	NS

### NF-κB is responsible for the initial expression of NLRP3 and the following dependent expression of IL-1β and BDNF

**Figure 4:**
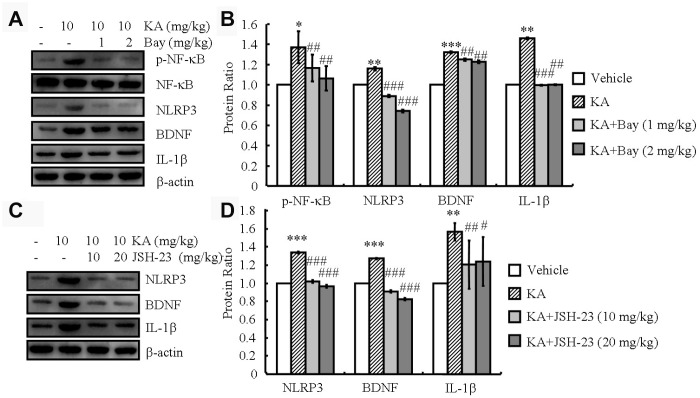
**Inflammasome mediates the KA-induced phosphorylation of NF-κB and the expression of NLRP3, IL-1β, and BDNF.** (**A** and **B**) Phosphorylation levels of NF-κB and expression levels of NLRP3, IL-1β, and BDNF in KA and/or Bay11-7082 in the brains of APP23 mice by Western blotting. The KA group was given i.p. injections of 10 mg/kg KA. The Bay11-7082+KA group mice were given i.p. injections of 1 or 2 mg/kg Bay11-7082 for 48 h (N=6). (**C** and **D**) The expression levels of NLRP3, IL-1 β, and BDNF in KA and/or JSH-23 in the brain of APP23 mice by Western blotting. The KA group was given i.p. injections of 10 mg/kg KA. The JSH-23+KA group mice were given i.p. injections of 10 or 20 mg/kg JSH-23 for 48 h (N=6). The optical density of bands in Western blotting was analyzed by ImageJ software ^*^P<0.05; ^**^P<0.01; ^***^P<0.001 vs the control group; ^#^*P*<0.05; ^##^*P*<0.01; ^###^*P*<0.001 vs the KA-only group.

The activity of the NLRP3 inflammasome is tightly regulated by NF-κB [16]. Consistently, in our analyses, the phosphorylation of NF-κB and the expression of NLRP3, IL-1β, and BDNF increased in the KA-treated mice and reached the highest level at 48 h (Figure 1A and 1B). Then, we treated the mice with KA (10 mg/kg) in the absence and presence of the inflammasome inhibitor Bay11-7082 (1 or 2 mg/kg) for 48 h. With the addition of Bay11-7082, the phosphorylation of NF-κB and the expression levels of NLRP3, BDNF, and IL-1β significantly decreased (Figure 4A, 4B), indicating that KA treatment can effectively induce the expression of IL-1β and BDNF via inflammasome activation. Considering the important role of the inflammasome in activating microglia, we further determined the effects of Bay11-7082 on the expression of CD68, a microglial marker reflecting the pathological tissue damage in the brains of APP23 mice [19]. The results demonstrated that Bay11-7082 decreased the protein levels of CD68 in the brains of APP23 mice (Supplementary Figure 1A), which is supported by previous studies showing that Bay11-7082 treatment attenuated the immunoreactivity of CD68 in the microglial cells of C57BL/6 mice [20]. To further verify the important roles of NF-κB in NLRP3, the NF-κB p65 [21] inhibitor JSH-23 (10 or 20 mg/kg) was administered to KA (10 mg/kg)-treated APP23 mice. JSH-23 inhibited the expression of NLRP3 (Figure 4C and 4D). In addition, the expression of IL-1β and BDNF was decreased by the administration of JSH-23 to the KA-treated mice (Figure 4C and 4D), indicating the functional role of NF-κB in NLRP3 regulation and the subsequent dependent expression of IL-1β and BDNF.

### IL-1β shows addictive whereas BDNF shows antagonistic effects of KA on inducing the expression of BACE1

**Figure 5:**
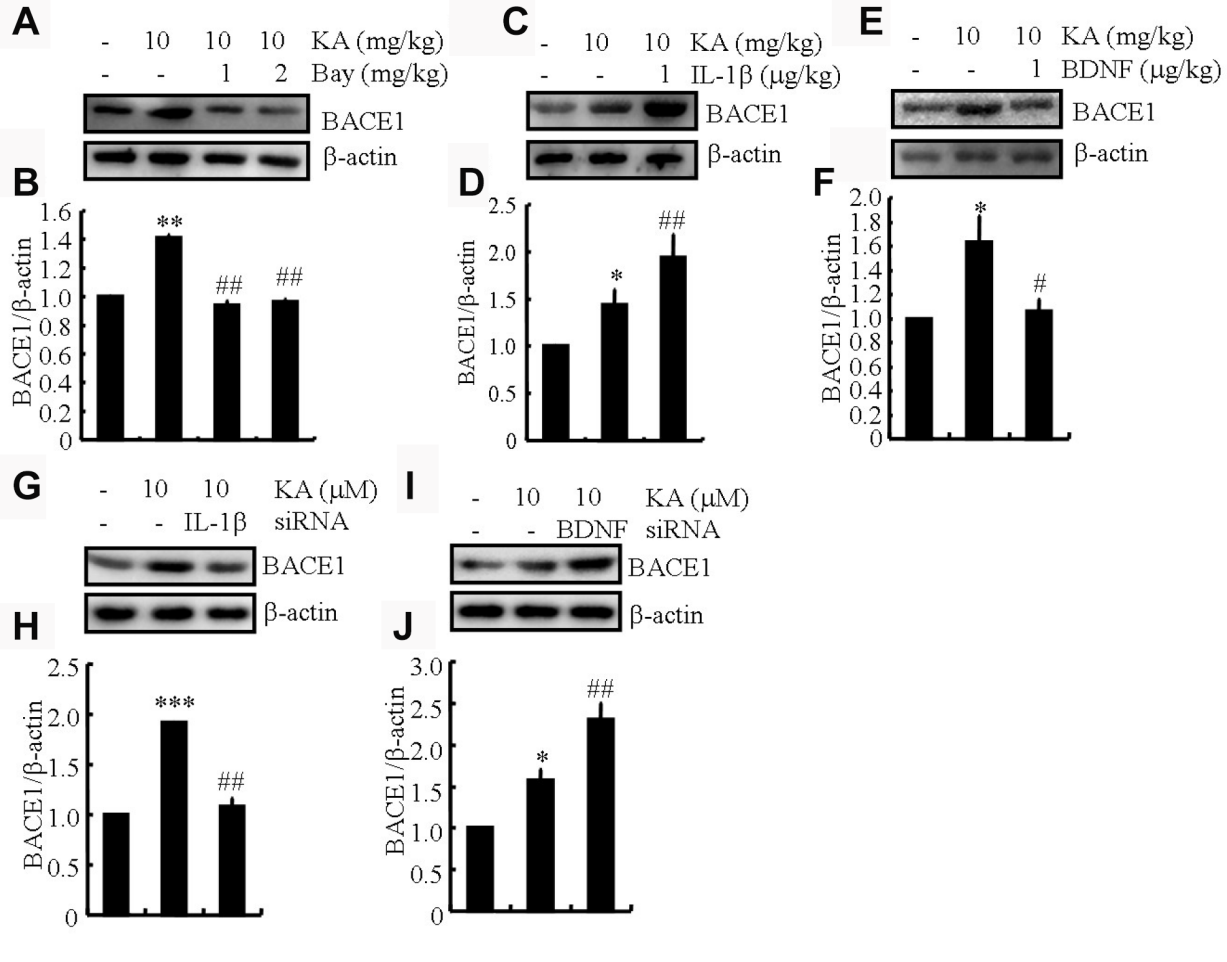
**IL-1β shows addictive effects whereas BDNF shows antagonistic effects of KA on inducing the expression of BACE1.** (**A** and **B**) Protein levels of BACE1 in the brains of KA (10 mg/kg)- or Bay11-7082 (1 or 2 mg/kg)+KA-treated APP23 mice (N=6). (**C** and **D**) Protein levels of BACE1 in the brains of KA- or IL-1β (1 μg/kg)+KA-treated APP23 mice (N=6). (**E** and **F**) Protein levels of BACE1 in the brains of KA- or BDNF (1 μg/kg)+KA-treated APP23 mice (N=6). (**G** and **H**) IL-1β and (**I** and **J**) BDNF were knocked down in the BV2 cells and the conditional medium was collected for the incubation of N2a cells. Protein levels of BACE1 were determined by Western blotting (N=3). The optical density of bands in Western blotting was analyzed by ImageJ software ^*^P<0.05, ^**^P<0.01, and ^***^P<0.001 vs the controls; ^#^*P*<0.05; ^##^*P*<0.01 vs the KA group; the significant differences from the respective values were determined by the one-way analysis of variance test.

BACE1 is an important secretase in AD-like Aβ production [22]. Our data show that KA plays an essential role in activating inflammasomes (Figure 4). Accordingly, we posited that KA might contribute to the abnormal cleavage of amyloid β-protein precursor protein (APP). In parallel with inflammasome biomarker changes, BACE1 expression was significantly upregulated in the KA-treated mice (Figure 5A and 5B), suggesting that the KA-induced inflammasome modulates BACE1 expression. To further verify the foregoing hypothesis, the mice were treated with Bay11-7082 (1 or 2 mg/kg) in the presence of KA (10 mg/kg). The results demonstrated that Bay11-7082 significantly attenuated the upregulation of BACE1 in the KA-treated mice (Figure 5A and 5B), indicating that the inflammasome acts upstream of BACE1 in the process of abnormal cleavage of APP and production of Aβ. To further assay the requirement of IL-1β and BDNF in KA-induced BACE1 expression, we applied IL-1β (1 μg/kg) and BDNF (1 μg/kg) in the KA-treated APP23 mice and found that IL-1β showed additive effects whereas BDNF showed antagonistic effects on the expression of BACE1 (Figure 5C–5F). To further validate the aforementioned results, IL-1β and BDNF were knocked down in the BV2 cells and the conditional medium was collected for incubation of N2a cells. The results demonstrated that IL-1β shows additive effects whereas BDNF shows antagonistic effects on the expression of BACE1 (Figure 5G–5J). Taken together, the results of our biochemical analyses suggest that inflammasome-dependent IL-1β activation underlies KA-induced BACE1 expression.

### Bay 11-7082 mitigates KA-induced production and aggregation of β-amyloid protein

**Figure 6:**
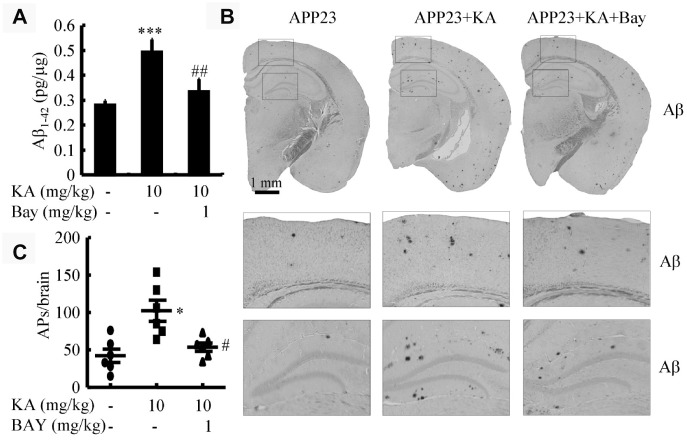
**Bay11-7082 suppresses KA-induced Aβ in the brains of APP23 mice.** (**A**) The production of Aβ in the brains of KA (10 mg/kg)- and/or Bay11-7082 (1 mg/kg)+KA-treated APP23 mice. (**B**) Immunohistochemical staining of Aβ in KA and/or Bay11-7082+KA-treated APP23 brains. (**C**) The visible number of APs in the brains of APP23 mice treated with or without KA and/or Bay11-7082+KA. ^*^P<0.05, ^***^P<0.001 vs controls; ^#^*P*<0.05, ^##^*P*<0.01 vs the KA group; the significant difference from the respective values were determined by the one-way analysis of variance test; N=6.

The aforementioned results suggest that KA-induced activation of inflammasomes triggers BACE1 expression. To determine whether KA increases or mitigates the production and aggregation of Aβ via inflammasomes, we assayed the production of Aβ after KA treatment (10 mg/kg) for 3 months in the absence and presence of Bay11-7082 (1 mg/kg) in 6-month-old APP23 mice. As shown, in the KA-treated groups, the production of Aβ significantly increased (Figure 6A). This is because of the inhibition of BACE1 in the APP23 mice (Supplementary Figure 1B). However, after the addition of Bay11-7082, the production of Aβ significantly decreased (Figure 6A). Consistently, the aggregation of Aβ in KA-treated mice could be significantly decreased by Bay11-7082 treatment (Figure 6B and 6C). On the basis of these findings, it is worth noting that inflammasome activation was the key cause of Aβ deposition in the KA-treated mice.

### KA impairs the learning ability of APP23 mice via inflammasome activation

**Figure 7:**
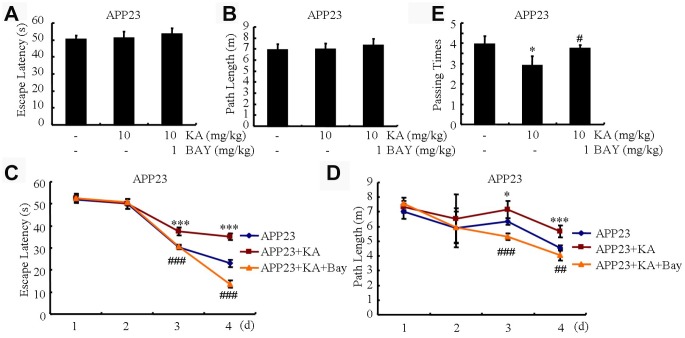
**Bay11-7082 mitigates KA-induced memory deficits using the Morris water maze test.** (**A** and **B**) During the first 2 days of visible platform tests, the KA and Bay11-7082 treated and control APP23 mice exhibited a similar latency to escape onto the visible platform. *P*>0.05 by Student’s t-test. (**C** and **D**) In the hidden platform tests, KA-treated APP23 mice showed a longer latency and length to escape onto the hidden platform on the third and fourth days, which was ameliorated by the addition of Bay11-7082 on the fourth day. ^*^P<0.05; ^***^P<0.001 vs the control group; ^##^*P*<0.01; ^###^*P*<0.001 vs the KA-treated group by ANOVA. (**E**) In the probe trial on the seventh day, the KA-treated APP23 mice traveled into the third quadrant, where the hidden platform was previously placed, significantly fewer times than the controls, which were improved by the Bay11-7082 treatment. ^*^P<0.05 vs the control group; ^#^*P*<0.05 vs the KA-treated group by ANOVA; N=6.

Memory impairment is closely associated with Aβ production and aggregation [23]. We speculated that Aβ alleviation by inhibiting the activity of inflammasomes might underlie the protective effect of Bay11-7082. To assess whether Bay11-7082 could alleviate KA-induced memory impairment, we conducted the MWM test to assay the learning ability of the mice. Within the first 2 days of tests, the mice showed a similar tendency to the visible platform, suggesting that the mice do not exhibit any defects or diseases (Figure 7A and 7B). We observed that the mean escape latency in the KA-only group increased relative to the control group and the escape latency of the KA+Bay11-7082 group was also shorter than that of the KA-only group (Figure 7C). Consistently, the path length was shorter in the KA+Bay11-7082 group than in the KA-treated group (Figure 7D). On the seventh treatment day, we removed the platform and conducted the probe trial. The platform-crossing frequency of the KA+Bay11-7082 group was higher than that of the KA-only group (Figure 7E). Taken together, these results demonstrated that Bay11-7082 ameliorates KA-induced memory deficits.

## DISCUSSION

**Figure 8:**
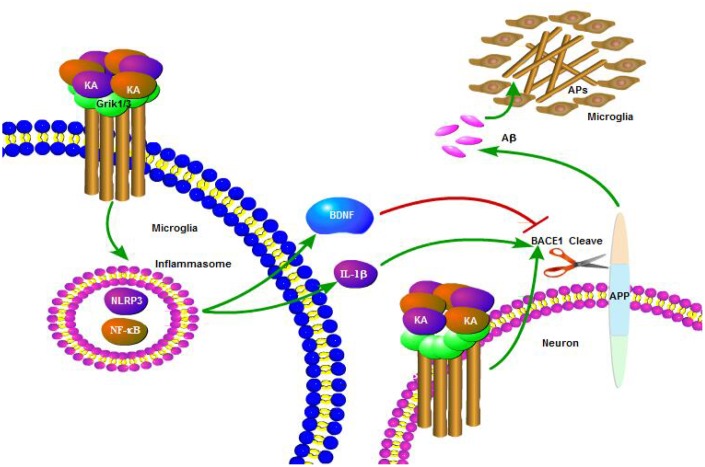
**A functional model of KA-induced activity of inflammasome that exacerbates Aβ production, deposition, and memory deficits.** KA treatment triggered the activation of inflammasome and caused the phosphorylation of NF-κB, leading to NLRP3 upregulation in microglia cells. Upregulated NLRP3 eventually resulted in the cleavage of APP by enhancing the expression of BACE1. Bay11-7082 inhibited the KA-induced IL-1β activation and Aβ production and deposition via alleviating the activity of inflammasome in neurons, which ultimately improved the cognitive decline of the APP23 mice.

The abnormal cleavage of APP and production of Aβ are the major components of APs, which positively correlate with the decline in memory and cognition in AD patients [24]. Multiple factors have been reported to play important roles in the etiology of AD in relation to its initiation and development. Excitation might occur in the early stages of AD and contribute to the neurodegenerative process [25]. The current study demonstrated that KA treatment induced inflammasome activity and activated NF-κB and NLRP3. Moreover, we showed that KA-induced inflammasomes significantly activated IL-1β and BDNF. In addition, IL-1β rather than BDNF is responsible for activating BACE1 and results in the production of Aβ (Figure 8). Importantly, we found that Bay11-7082 can appreciably ease KA-induced production and aggregation of Aβ and the cognition disabilities by manipulating the inflammasome-mediated signaling pathway.

The inflammasome has been shown to contribute to various disorders in the central nervous system [13]. In AD brains, the extracellular accumulation of Aβ in APs is the featured principal event in AD pathogenesis [24]. The deposition of Aβ peptide initiates inflammasome activity in microglia [14]. More interestingly, inflammasome activation plays a role in the impairments related to AD pathogenesis, such as Aβ deposition and loss of spatial memory, by mediating a harmful chronic inflammatory response [13]. The inflammasome, belonging to the NLR families, is a multi-protein signaling platform that detects microbial components, endogenous stress, and damage signals in intracellular environments [26]. To date, several inflammasome complexes have been well characterized and are typically named after their NLR protein (NLRP) [27]. Among NLRPs, NLRP3 inflammasomes have been reported to participate in several chronic inflammatory and autoimmune diseases [28]. For AD, the levels of multiple mediators, including cytokines and chemokines such as IL-1β, are elevated in the cerebrospinal fluid (CSF) and post-mortem brain tissues of patients with a history of neuroinflammatory conditions [29, 30]. High levels of cytokines and chemokines secreted from microglia and astrocytes are a consistent indicator of the pathogens and other inflammatory triggers that could participate in the assembly and activation of inflammasomes. The aforementioned process could ultimately drive the caspase-1-mediated maturation of IL-1β cytokines [31]. Although a series of investigations have revealed the expression of IL-1β in microglia surrounding APs in AD [32, 33], Halle et al. first described NLRP3 inflammasome activation by Aβ in microglia in AD, which resulted in the maturation and secretion of IL-1β [14]. More interestingly, the activation of the NLRP3 inflammasome further promoted the synthesis of cytokines and chemokines by microglia, which results in the aggregation of inflammatory and potentially neurotoxic factors, such as Aβ [13, 14, 34]. Therefore, it would be interesting to propose that Aβ deposition is not only a cause, but also a consequence of inflammasome activation in AD. Consistently, this is another research result suggesting that APs recruit and activate microglia, which induce the expression of pro- inflammatory molecules, influence surrounding tissues, and amplify the neurotoxic effects of Aβ [31]. In line with these studies, our data further showed that KA is a neurotoxic factor activating inflammasomes in the process of AD.

Brain NLRP3 activation was restricted to plaque-associated microglia [13], which suggests that microglial activation of the NLRP3 inflammasome is pivotal for AD pathogenesis. Actually, it is already accepted that the pathogenesis of AD correlates with local neuroinflammation. For instance, deregulation and alternation of microglial activity have been reported to induce the pathway toward neurodegeneration in AD [35]. The excessive loading of inflammatory cytokines and chemokines from microglia may induce signaling events in neurons and ultimately result in damage to brain tissue [36]. More specifically, IL-1β has been identified to exacerbate AD pathogenesis through tau phosphorylation [37], which impairs the learning and memory of AD mice [38, 39]. Additionally, blocking the signaling pathway of IL-1β in vivo provided disease-modifying benefits in an AD experimental model [40]. In cultured rat cortical neurons, both IL-1β and IL-6 played important roles in Aβ deposition [41]. In agreement with these reports, the current study further shows that inflammasomes promoted the “downstream” microglial synthesis of IL-1β, which contributes to the production and aggregation of Aβ. The secretion of IL-1β is just one of the effects of NLRP3 inflammasome activation, and the mechanisms underlying inflammasome-dependent pyroptosis in AD remain underexplored at present. In addition to IL-1β, BDNF was also induced by NLRP3 inflammasome activation (Figure 4). Relative to IL-1β, BDNF exerts neuroprotective effects by reducing the expression of BACE1 (Figure 5). Indeed, BDNF has shown neuroprotective effects against glutamate-induced apoptotic cell death in cultured hippocampal neurons [42]. However, the protective effects of BDNF could not diminish the impairing roles of IL-1β in AD since the ultimate effects of KA were exacerbating Aβ deposition and impairing the learning and memory of APP23 mice.

As previously discussed, the potential roles of the NLRP3 inflammasome in the pathogenesis of AD provide novel insights into the inflammasome signaling pathway in AD. NLRP3 knockout significantly suppressed amyloidosis and neuropathology and alleviated the cognitive decline in AD experimental animals [13]. Moreover, the inhibition of NF-κB and NLRP3 inflammasome activation attenuated neuroinflammation and amyloidogenesis [43]. Therefore, the NLRP3 inflammasome could become a potential molecular target for neuroprotection and therapeutic intervention for AD. Development of agents to critically control the activation of the NLRP3 inflammasome at the molecular level might have considerable promise to suppress neuroinflammation and delay the onset and progression of AD. For this reason, our data showed that the NLRP3 inflammasome inhibitor Bay11-7082 blocked the stimulating effects of KA on the production and deposition of Aβ as well as the cognitive decline in APP23 mice (Figure 6). These observations also indicate the potential therapeutic effects of Bay11-7082 against KA in AD via deactivation of the inflammasome.

## MATERIALS AND METHODS

### Mice and treatment protocol

APP23 [B6-Tg(Thy1APP)23SdZ] mice were obtained from the Jackson Laboratory (Stock #030504). The generation of the mouse line APP23, which is transgenic for human amyloid precursor protein (APP) carrying the Swedish mutation, has been described elsewhere [44]. All animal procedures were approved by the Institutional Animal Care and Use Committee of the First Hospital of Jilin University in compliance with the Guidelines for the Care and Use of Laboratory Animals of the U.S. National Institutes of Health. The mice were housed five per cage in a room maintained at 22 ± 2°C with an alternating 12-h light-dark cycle. Food and water were available ad libitum.

Based on a previous study, a total of 6 male mice were treated with an intraperitoneal (i.p.) injection of 10 mg/kg KA (Sigma-Aldrich Corp, St. Louis, MO, USA) emulsified in 0.9% PBS (-). To validate the neurotoxicity induced by KA, behavioral observations were conducted every 30 min for 4 h after KA injection. The mice that did not display general limbic seizure activity within 90 min after the KA injection were excluded from the study. The male mice in the control group (n = 6) were injected with PBS (-). The animals in the experimental group were sacrificed 6, 12, 24, 48, and 96 h after KA treatment.

In short-term treatment assessments, the male mice were randomly divided into three to four groups, each containing 6 mice: a KA-only group (10 mg/kg); a group receiving Bay11-7082 (1 or 2 mg/kg) (Sigma-Aldrich Corp, St. Louis, MO, USA), JSH-23 (10 or 20 mg/kg) (Sigma-Aldrich Corp, St. Louis, MO, USA), IL-1β (1 μg/kg) (Thermo Fisher Scientific, Shanghai, China), and BDNF (1 μg/kg) (Sigma-Aldrich Corp, St. Louis, MO, USA) administration with KA (10 mg/kg); and a vehicle-treated control group (control). In long-term treatment assessments, 3-month-old mice were similarly treated with KA (10 mg/kg) or Bay11-7082 (1 mg/kg)/KA per day for 3 months before the Morris water maze (MWM) test and brain collection. All of the mice were sacrificed after treatment, and the brain tissue was harvested for further tests.

### Morris water maze test

To assess the cognitive changes, the MWM test was conducted in a circular water tank (1.4 m in diameter and 40 cm in height) filled with water to a depth of 20 cm and maintained at 21 ± 1°C. The tank was divided into four equal quadrants. A submerged square platform was placed in the third quadrant of the tank with its top surface 1 cm below the water surface. The mice were placed in the pool at four possible start locations facing the wall of the pool, and a camera was simultaneously activated. Each mouse was allowed up to 60 s to locate the platform. The trial was terminated when the mouse found the platform within 60 s. If the mouse failed to find the platform within 60 s, it was guided by a researcher to locate the platform and allowed to stay there for 2-3 s. Each mouse was conditioned three times per day for 2 days to allow it to adapt to the pool environment (visible platform training) and then tested 3 times per day for 4 days to find the hidden platform (hidden platform training). The latency (the time taken to locate the platform in the water), distance, and swimming speed were recorded using an automated video-tracking software package (Noldus EthoVision 2.3.19, Wageningen, Netherlands). On the seventh day, the platform was removed, and the number of times the mouse passed the original location of the platform was tracked and recorded on video.

### Immunochemistry

Mouse brain tissues were frozen and sectioned consecutively at 10 μm, with three sections for each embedded sample. The sections were incubated overnight at 4°C with Aβ antibody (1:500, Cell Signaling Technology, Danvers, MA, USA). After incubation with horseradish peroxidase-conjugated secondary antibody (Cell Signaling Technology, Danvers, MA, USA) for 2 h at room temperature, the sections were washed and developed with 0.05% diaminobenzidine (DAB) plus 0.015% hydrogen peroxide in PBS. The DAB-stained sections were air-dried, counterstained with Mayer’s hematoxylin, dehydrated, cleared, and coverslipped. Finally, the sections were visualized with DAB for light microscopy examination (Olympus BX50, Center Valley, PA, USA).

### Double immunofluorescent staining

Frozen sections (thickness, 10 μm) of the mice brain were blocked with 5% normal goat serum in TBS for 30 min, followed by incubation with primary antibodies overnight at 4°C in TBS containing 5% goat serum and 0.1% Triton. Primary antibodies used included mouse monoclonal anti-Aβ (1:250), rabbit monoclonal anti-NF-κB p65 (1:50, Cell Signaling Technology, Danvers, MA, USA), and rabbit monoclonal anti-NLRP3 (1:100, Cell Signaling Technology, Danvers, MA, USA). The antibody used for NF-κB in the following experiments was probing the p65 subunit. After washing with TBS, the sections were incubated with anti-mouse IgG (H+L), F(ab')_2_ fragment (Alexa Fluor 488 Conjugate) (1:1000, Cell Signaling Technology, Danvers, MA, USA) and anti-rabbit IgG (H+L), and F(ab')_2_ fragment (Alexa Fluor 594 Conjugate) (1:1000, Cell Signaling Technology, Danvers, MA, USA) in TBS containing 5% goat serum and 0.1% Triton at room temperature for 1 h. The double immunostaining was analyzed using a laser scanning confocal microscope (A1, Nikon, Shanghai, China).

### BV2 and N2a cell culture and RNA interference

BV2 cells were cultured in a humid incubator with 95% air and 5% CO_2_ at 37°C. The DMEM medium (Gibco, Grand Island, NY, USA) with 10% FBS (Gibco, Grand Island, NY, USA) was changed when the color turned yellow. In the experiments, the BV2 cells were pre-transfected with or without siRNAs targeting Grik1-3, KA1-2, IL-1β, or BDNF (200 ng, Santa Cruz Biotechnology, Shanghai, China) using FuGENE 5 transfection reagent (Roche Diagnostics, Indianapolis, IN, USA) according to the manufacturer’s instructions. After 48 h, the cells were stimulated with KA (10 μM). Corresponding dilutions of DMSO were added to the control cells. To determine the effects of IL-1β and BDNF on the expression of BACE1, IL-1β and BDNF were knocked down in the BV2 cells and the conditional medium was collected for incubation of N2a cells.

### Western blotting

Total proteins were extracted using a protein extraction kit (Thermo Fisher Scientific, Shanghai, China) following the manufacturer’s protocol. Protein extracts were dissolved in 10%–15% SDS-PAGE and then transferred to a PVDF membrane at 100 V for 1 h. After blocking with 5% non-fat skim milk diluted with Tris-buffered saline containing 0.1% Tween 20 (TBST) for 1 h at room temperature, the membrane containing the protein extracts was incubated overnight with primary antibody (diluted with 2% bovine serum albumin in TBST) at 4°C. The following primary antibodies were used: anti-β-actin (1:5000, Cell Signaling Technology, Danvers, MA, USA); anti-p-NF-κB (1:2000, Cell Signaling Technology, Danvers, MA, USA); anti-NF-κB (1:2000, Cell Signaling Technology, Danvers, MA, USA); anti-NLRP3 (1:3000, Cell Signaling Technology, Danvers, MA, USA); anti-IL-1β (1:1000, Cell Signaling Technology, Danvers, MA, USA); anti-BDNF (1:1000, Cell Signaling Technology, Danvers, MA, USA); and anti-BACE1 (1:2000, Cell Signaling Technology, Danvers, MA, USA). On the second day, the proteins were visualized using an enhanced chemiluminescence detection system (Thermo Fisher Scientific, Shanghai, China) after incubation with the corresponding secondary antibodies (1:10000, Cell Signaling Technology, Danvers, MA, USA) and visualized using Bio-Rad ChemiDocXRS devices (Bio-Rad Laboratories, Shanghai, China). For quantitative analysis of the band intensity, we calculated the band intensity ratio for normalization using ImageJ software. For each blot, we multiplied the background subtracted density of the target protein in each lane by the ratio of the density of the loading control (such as a housekeeping protein) from a control sample of all of the study blots to the other lanes in the gel. This procedure yielded the normalized density to the loading control (NDL). We calculated the fold difference for each replicate by dividing the NDL from each lane by the NDL from the control sample.

### Enzyme activity assay

For sandwich ELISA, a 96-well plate (MaxiSorp, Nunc, Denmark) was coated with 100 μl of Aβ 42-specific antibody (5.0 μg IgG/mL each) overnight at 4°C in 100 mM carbonate buffer, pH 9.6, containing 0.05% sodium azide. After blocking with 1% Block Ace in PBS overnight at 4°C, the standards (human synthetic Aβ peptides 1–42) and samples were loaded and incubated overnight at 4°C. HRP-coupled detection antibody was incubated for 4 h at room temperature and visualized using a TMB substrate.

### Statistical analysis

The data were expressed as mean ± standard deviation and analyzed using SPSS 10.0 statistical software (SPSS Inc., Chicago, IL, USA). One-way and two-way analysis of variance (ANOVA) tests were used to determine the significance of the differences among groups (*P* < 0.05, *P* < 0.01, and *P* < 0.001).

## Supplementary Material

Supplementary Figure
