# Supplementary material for: Kainic acid Induces production and aggregation of amyloid β-protein and memory deficits by activating inflammasomes in NLRP3- and NF-κB-stimulated pathways
Source: Aging (Albany NY). 2019 Jun 10;11(11):3795–810. doi: 10.18632/aging.102017 (PMC6594814; doi:10.18632/aging.102017)
Supplement: Supplementary Figure [file aging-11-102017-s001.pdf]

SUPPLEMENTARY FIGURE

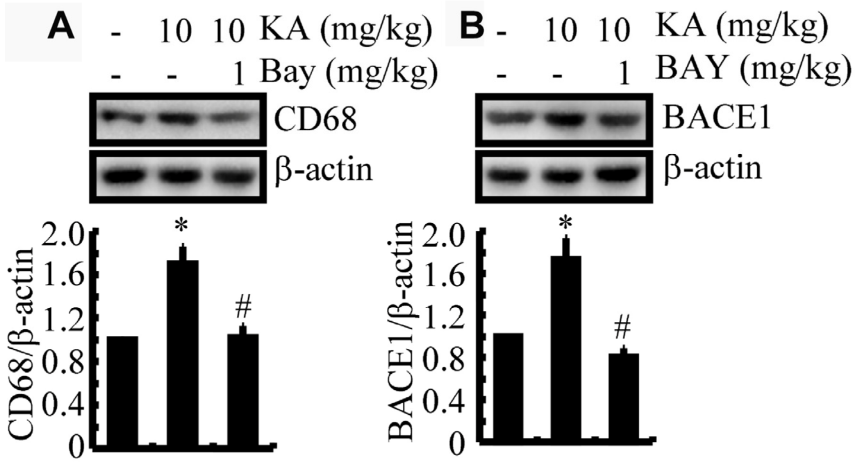

Supplementary Figure 1. Bay11-7082 attenuates KA-induced the expression of CD86 and BACE1. (**A**) The expression of CD68 in the brains of KA (10 mg/kg)- and/or Bay11-7082 (1 mg/kg)+KA-treated APP23 mice. (**B**) The expression of BACE1 in the brains of KA (10 mg/kg)- and/or Bay11-7082 (1 mg/kg)+KA-treated APP23 mice. The optical density of bands in western blots was analyzed by image J software (* $P < 0.05$ vs. controls; # $P < 0.05$ vs. the KA group; the significant difference from the respective values were determined by one-way analysis of variance test, N=6).
